# Bridging Animal and Human Data in Pursuit of Vaccine Licensure

**DOI:** 10.3390/vaccines10091384

**Published:** 2022-08-25

**Authors:** Courtney L. Finch, William E. Dowling, Thomas H. King, Christian Martinez, Bai V. Nguyen, Ramon Roozendaal, Roxana Rustomjee, Mario H. Skiadopoulos, Ekaterina Vert-Wong, Ann Yellowlees, Nancy J. Sullivan

**Affiliations:** 1 Sabin Vaccine Institute, Washington, DC 20037, USA; 2 Coalition for Epidemic Preparedness Innovations, Washington, DC 20006, USA; 3 Biomedical Advanced Research and Development Authority, Office of the Assistant Secretary for Preparedness and Response, Department of Health and Human Services, Washington, DC 20201, USA; 4 Janssen Vaccines and Prevention B.V., Leiden Archimedesweg 4, 2333 CN Leiden, The Netherlands; 5 Microvirion Vaccine Consulting, Rockville, MD 20852, USA; 6 Quantics Biostatistics, Edinburgh EH3 8EG, UK; 7 Vaccine Research Center, National Institute of Allergy and Infectious Diseases, National Institutes of Health, Bethesda, MD 20892, USA

**Keywords:** immune correlate, immunobridging, Animal Rule, ELISA, PsVNA, binding, neutralization, animal model, vaccine, filovirus

## Abstract

The FDA Animal Rule was devised to facilitate approval of candidate vaccines and therapeutics using animal survival data when human efficacy studies are not practical or ethical. This regulatory pathway is critical for candidates against pathogens with high case fatality rates that prohibit human challenge trials, as well as candidates with low and sporadic incidences of outbreaks that make human field trials difficult. Important components of a vaccine development plan for Animal Rule licensure are the identification of an immune correlate of protection and immunobridging to humans. The relationship of vaccine-induced immune responses to survival after vaccination and challenge must be established in validated animal models and then used to infer predictive vaccine efficacy in humans via immunobridging. The Sabin Vaccine Institute is pursuing licensure for candidate filovirus vaccines via the Animal Rule and has convened meetings of key opinion leaders and subject matter experts to define fundamental components for vaccine licensure in the absence of human efficacy data. Here, filoviruses are used as examples to review immune correlates of protection and immunobridging. The points presented herein reflect the presentations and discussions during the second meeting held in October 2021 and are intended to address important considerations for developing immunobridging strategies.

## 1. Introduction

An immune correlate of protection is defined as an immune marker that is associated with protection from clinical disease. Immunobridging of animal immunogenicity and survival data to human immunogenicity data via an immune correlate of protection is critical in inferring predictive human vaccine efficacy from animal data [1,2,3]. Such a prediction becomes essential when human efficacy data are unavailable.

While it is relatively uncommon to obtain vaccine licensure without human efficacy data, there are regulatory pathways that allow for it [1,4,5]. Perhaps the most formal pathway for such a mechanism is the United States (U.S.) Food and Drug Administration (FDA) Animal Rule (Animal Rule) [1,6]. The concepts we address here are generalizable and important for inferring predictive human efficacy from animal data, but they are also framed specifically around the Animal Rule. 

The Animal Rule is not a shortcut to licensure or a means to circumvent lengthy and costly human efficacy trials. Instead, it provides an alternative to traditional licensure pathways when human challenge trials (i.e., deliberate exposure of humans to the pathogen per an approved clinical trial protocol) are not possible due to the high lethality of the pathogen, or when human field trials (i.e., natural exposure of humans to the pathogens during an outbreak scenario) are impractical due to the infrequent and unpredictable incidence of disease outbreaks, prohibiting the collection of adequate human efficacy data. Although the Animal Rule does not require human efficacy data, other traditional pathway regulatory requirements, such as the demonstration of safety and immunogenicity in humans through clinical trials, are required to support licensure [1]. To use the Animal Rule pathway for vaccine licensure (21 CFR 314.600–314.650 and 21 CFR 601.90–601.95), several elements may apply (with the caveat that the Animal Rule focuses primarily on vaccines):

1. “There is a reasonably well-understood pathophysiological mechanism of the toxicity of the substance and its prevention or substantial reduction by the product;
2. The effect is demonstrated in more than one animal species expected to react with a response predictive for humans, unless the effect is demonstrated in a single animal species that represents a sufficiently well-characterized animal model for predicting the response in humans;
3. The animal study endpoint is clearly related to the desired benefit in humans, generally the enhancement of survival or prevention of major morbidity; and
4. The data or information on the kinetics and pharmacodynamics of the product or other relevant data or information, in animals and humans, allows selection of an effective dose in humans” [1] (Sec. 314.610). (In the case of vaccines, immune responses are the relevant parameter, as pharmacodynamics and kinetics are not applicable) [7].

Filovirus vaccines are ideal examples likely to require use of the Animal Rule pathway to licensure, due to the low and sporadic incidences of outbreaks and high case fatality rates. Filoviruses are members of the family Filoviridae [8]. Within this family, there are eight genera and at least six viruses that are known to infect humans [8,9,10,11,12,13,14]. The viruses responsible for most human outbreaks are Ebola virus (EBOV), Marburg virus (MARV) and Sudan virus (SUDV) [11]. Human infections are the result of zoonotic, animal-to-human transmission, or human-to-human transmission via contact with virus-infected bodily fluids and tissues [15,16]. Manifestations of disease related to infection with these three viruses are generally similar; case fatality rates are high (for example, EBOV case fatality ranges from about 25–90%). Virus outbreaks tend to be small and infrequent although large outbreaks have occurred, specifically in the case of EBOV [15,16,17,18]. The incubation period is 2 to 21 days; the specific incubation time may depend on several factors such as the virus species, the manner of exposure and the virus exposure dose [17]. Disease presentation begins with fever, myalgia and fatigue that may progress to gastrointestinal symptoms such as nausea, vomiting and diarrhea. Headache, maculopapular rash and abdominal pain are other common manifestations. Bleeding abnormalities, which have previously been falsely perceived as occurring in most cases, occur but in a minority of cases [17]. Ultimately, infection spreads throughout the body, infecting multiple organs, especially the liver and spleen, and often leads to multiorgan failure and death [17]. The cellular response to infection is dynamic and may include, but is not limited to, robust proinflammatory cytokine and chemokine responses, T-cell activation and bleeding/coagulopathy caused in part by functional inhibition of platelets [17]. 

Filoviruses are negative-strand RNA viruses containing up to 19 kb genomes, most with seven genes, including glycoprotein (GP), which encodes the surface GP, the target antigen used in vaccines currently in development [12,19,20,21]. Many filovirus vaccines are gene-based and incorporate GP into a modified, less pathogenic virus as a vector to deliver GP [21]. GP is an ideal candidate antigen for vaccine development because it is highly immunogenic and mediates the first step in virus replication [22,23]. Anti-GP antibody has been defined as a vaccine-induced immune correlate of protection (although there are nuances to this conclusion that are discussed below), and GP-directed monoclonal antibodies have been shown to reduce mortality caused by Ebola virus disease in humans. ERVEBO, a GP-based EBOV vaccine, induces anti-GP antibodies and is efficacious in humans at preventing disease [2,24,25,26,27]. 

The Sabin Vaccine Institute (Sabin) is pursuing licensure for vaccine candidates against two filoviruses, MARV and SUDV. Due to the complexity and novelty of vaccine licensure via the Animal Rule being sought by Sabin, a key opinion leader (KOL) meeting series was convened with experts in the field to discuss critical aspects of vaccine licensure in the absence of human efficacy data. Few vaccines have been approved without human efficacy data, but rare examples exist. These include BioThrax anthrax vaccine adsorbed (AVA) in the U.S. and Zabdeno, Mvabea in the European Union and in select African countries [5,28]. With so few precedents, there is still much to learn to advance vaccines using the Animal Rule, and the goal of the Sabin KOL series is to identify critical steps that can be applied across vaccine platforms. 

The first KOL series meeting focused on vaccine regulatory requirements in the U.S. and abroad, and the importance of the alignment of regulatory pathways that allow for licensure in the absence of human efficacy data [29]. This review will summarize key points from the second meeting entitled, “Blue Ribbon Panel: Immune Correlates of Filovirus Protection at the Human-Animal Interface, a Pathway to Licensure”, which focused on immune correlates of protection and immunobridging. 

Based upon KOL meeting discussions, herein we review the importance of animal model and immune correlate selection as they relate to immunobridging and provide two case studies and key considerations for planning immunobridging strategies in pursuit of licensure via the Animal Rule. We aim to provide a strong foundation for understanding the scientific components of immunobridging to infer human vaccine efficacy from animal data. We use filoviruses as example pathogens for these discussions, but these topics are relevant to other pathogens as well. The opinions and ideas presented here are those of the authors and are not intended to reflect the position of any of the institutions or organizations with which the authors are affiliated.

## 2. Animal Models and Immune Correlates of Protection 

The foundation of an effective immunobridging strategy begins with selection of the appropriate animal model(s) and the immune correlate(s) of protection (which is established in the animal model via efficacy studies). The immune correlates we discuss here are defined as immunological markers that correlate with protection and are either mechanistic (i.e., causally related to protection) or non-mechanistic (not causally related to protection) in nature. The first step in identifying an immune correlate of protection is to identify an animal model that reasonably predicts human pathogenesis and immune responses, and infection conditions that allow breakthrough in vaccine protection. Two animal models/species may be required according to Animal Rule guidelines if there is no single animal model that represents a sufficiently well-characterized animal model for recapitulating human disease, and the selected animal model(s) must be agreed upon by the FDA [1]. Animal model selection is so critical that three of the four Animal Rule criteria (criteria 2–4, described in Section 1 above) stipulate requirements of the animal model(s); the more similar the pathophysiology between animal and human, the more relevant to humans the immune correlate of protection defined in the animal model is considered to be [1]. 

There are numerous filoviral animal models of disease available with varying levels of relevance to human pathophysiology. Mouse, guinea pig and hamster models are cost-effective, but they have not been considered suitable models for Animal Rule licensure, in part due to the need for challenge virus adaptation and/or genetic attenuation of the immune system to yield severe disease [30,31,32,33,34,35,36,37]. In other words, efficacy against the human wild-type virus for which the candidate vaccine is ultimately intended cannot be directly evaluated in these models. Ferrets, in contrast, display more faithful mimicry of the pathophysiology of human disease (for example, EBOV and SUDV) compared to rodents without virus adaptation. Unfortunately, commercially available ferret-specific immune reagents are somewhat limited, and ferrets are not susceptible to MARV infection, complicating vaccine development and evaluation of multivalent vaccines [38,39].

For filoviruses such as EBOV, MARV and SUDV, nonhuman primates (NHPs) are considered the gold standard for evaluation of vaccine and therapeutic efficacy; rhesus and cynomolgus macaques are used most commonly [40,41]. When comparing filovirus disease between NHPs and humans, the constellation of clinical signs and the pathophysiology are similar [15,16,38,40,42,43,44,45]. No other filovirus animal model faithfully mimics human disease pathophysiology as well as NHPs [38,40,43,44]. This is not surprising given the genomic homology between NHPs and humans [46,47]. For instance, dysregulation of coagulation pathways, a key hallmark of human filovirus infection/pathogenesis, is observed in NHP models, but only a subset of these coagulopathies is present in mice [38,43,48]. 

Still, NHPs do not perfectly mimic human disease. The progression of filoviral disease in fatal NHP models is accelerated compared to the kinetics in humans, the entire disease course being up to about 13 days (depending on the filovirus, the animal species/model and other factors) compared to an incubation period in humans of up to 21 days (although the exact number of days is likely dependent on the factors discussed in the section above) [17,43,44]. Additionally, NHP filoviral models for EBOV, MARV and SUDV use a virus challenge dose and route that are nearly uniformly lethal, whereas human case fatality rates average roughly half that for most filoviruses (MARV, 24–88%; EBOV, 25–90%) [15,16,38,49,50,51,52,53,54]. Importantly, while there is a difference in lethality between humans and NHPs, ERVEBO showed 100% survival in NHPs, and that predicted high-level protection in a human efficacy trial [25,55,56]. High lethality in the NHP challenge does yield a stringent test of vaccine efficacy. Likewise, the intramuscular injection virus exposure route in NHPs aims for uniformity in the high dose exposure and has relevance to human needlesticks that resulted in fatalities [57,58]. Since the current review is focused on immune correlates of protection and immunobridging, we refer the reader to several comprehensive reviews for additional details of filoviral animal models [38,40,43,44].

Following identification of an animal model(s) for evaluation of a vaccine using the Animal Rule, an immune correlate(s) of protection must be identified where the immune correlate should be predictive of the desired study endpoint such as viral load or survival [1]. Correlates of protection may vary depending on the vaccine platform, antigen, its route of administration, virus species and the animal model [7,59]. Clinical trials must be done to confirm that the defined NHP immune correlate is assayable in humans and in the selected animal species/model in the same assay format; these correlates are not necessarily related to the mechanism of protection [1,7,59]. Immune correlates of survival associated with natural immunity may differ from those of vaccine-derived immune correlates of protection [7,60]. For some pathogens, defining the immune correlate of protection is relatively straightforward. For instance, protection against severe acute respiratory syndrome coronavirus 2 (SARS-CoV-2) has been shown to significantly correlate with neutralizing antibody titers, and as we will discuss below, toxin-neutralizing antibodies (TNA) are strongly predictive of survival against anthrax [61,62,63,64,65]. Importantly, these correlates need to be confirmed through properly designed animal studies and validated immunoassays.

Not every pathogen will have such clearly defined correlates of protection against disease. Vaccine-induced (and natural) protection against filovirus infection appears to be multifactorial and linked to both T cell (especially CD8^+^) responses and anti-filovirus GP immunoglobulin G and M (IgG and IgM, respectively) serum antibody responses [7,66,67,68,69,70,71,72,73,74]. While T cells play a mechanistic (causal) role in protection for a GP-based vaccine against Ebola virus, a statistically significant GP-specific T-cell correlate of protection is more difficult to define, due in part to the dynamic nature of T-cell responses [67]. For the same vaccine, passive transfer of vaccine-induced IgG failed to provide complete protection against filovirus infection in NHPs, suggesting that antibodies may not play a mechanistic role in protection [67]. In contrast, protection conferred by another GP-based vaccine, also against Ebola virus, but on a different platform, seems to be more dependent on antibodies, as CD4+ depletion in NHPs abrogated GP-specific antibody production and animals succumbed to virus challenge [69]. To date, there are no data to definitively support GP-specific antibodies as mechanistic correlate. Nonetheless, vaccine-induced antibodies do correlate with macaque survival in several studies [2,26], and studies indicate that anti-GP IgG correlates with human survival for ERVEBO [75]. Notably, passively infused GP-directed monoclonal and polyclonal antibody therapeutics have been shown to confer protection in both macaques and humans, thus demonstrating the biological relevance of the immune correlate [24,76,77]. The varied outcomes in these studies draw attention to the difference between correlation and causation with respect to the role of antibodies in protection against filoviral disease and emphasize the case-by-case (model-dependent) ascribed role of antibodies. 

Nearly as important as biological relevance in the selection of an immune correlate of protection are practical considerations. For example, T-cell assays appropriate to assess cellular immunity in a preclinical setting have been established; however, these assays can be difficult to implement and validate for clinical trials as they require more complex procedures than those used for antibody assessment. Additionally, T-cell responses are dynamic and transient [78]; capturing them to define a precise quantitative correlate of protection is difficult when compared to IgG antibodies, which are more stable and longer lasting. Not only are anti-filovirus serum GP IgG antibody levels easier to capture, but they are also comparatively easy to assess in enzyme-linked immunosorbent assays (ELISAs). Although total binding antibody titers (as assessed by ELISA) are not necessarily mechanistic correlates, they correlate well with protection in at least some studies [2,26]. Neutralizing antibody titers, such as those defined by plaque reduction neutralization assays and pseudovirus neutralization assays, have been shown to correlate with protection against mortality and have been identified in human filovirus disease survivors [2,79,80,81]. Neutralization assays, however, are more complicated (using live virus) than ELISAs and are less predictive of survival in some filovirus challenge studies [74,82]. Total serum binding anti-filovirus GP IgG titers are good overall correlates of protection (as defined in NHPs), both biologically and practically, due to the strength of correlation and the relative simplicity of measurement [2,7,26,83]. 

Such careful consideration given to selecting an immune correlate(s) of protection presupposes that the bioassay used to measure the correlation must be validated, robust and feasible in the clinic and the laboratory. Concurrence with the FDA on the immune correlate of protection to be evaluated during vaccine development is critical, since the immune correlate serves as the basis for inferring predictive human efficacy from animal immunogenicity and survival data. For further reading, filovirus immune correlates have been well reviewed elsewhere [7,59,72,84].

## 3. Overview of Immunobridging

Immunobridging is the process by which human vaccine efficacy is inferred based on animal immunogenicity and survival data. The immune correlate of protection should be assessed in humans and animals using the same assay. Below, we describe the basic approach to immunobridging using example data. 

The first step in the process is to define a statistically significant relationship between the candidate immune correlate of protection and survival. This involves a series of challenge studies in which animals are vaccinated at a range of doses with the aim of achieving antibody levels that span a range of survival rates. This is needed so that the probability of survival for a human subject with a given antibody level can be precisely predicted when the corresponding human antibody levels become available from clinical trials. This precision is driven by the approximation of the parameter estimates for the modeled relationship; this in turn is driven by both the size of the NHP data set and the range of antibody levels seen in the data across the range of protection. It is particularly important to collect data for antibody levels achieving intermediate protection, not only at the extremes of 0% and 100% survival.

**Figure 1:**
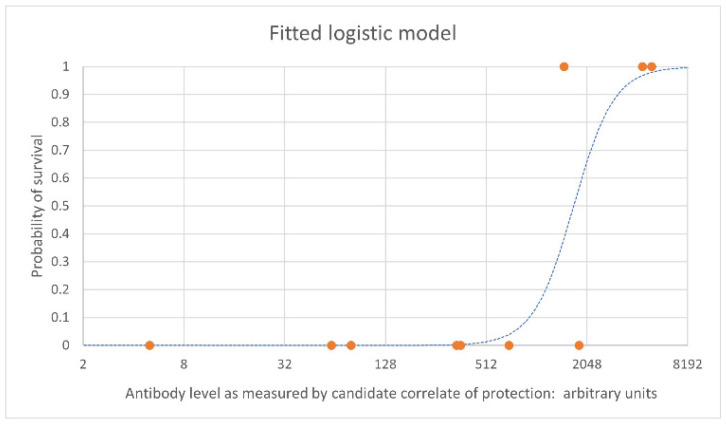
Example logistic regression curve. Example data is used here. The blue dashed line is the estimated logistic model which indicates the probability of survival. The orange points represent individual NHPs. NHPs that survive are shown at 1 on the y axis; NHPs that died are shown at 0 on the y axis.

Figure 1 shows an example of an estimated logistic relationship between vaccine-induced antibody levels pre-challenge (on the x axis) and the probability of survival at a given timepoint following challenge in NHPs (on the y axis). Each orange point represents individual NHPs that survived (at 1 on the y axis) and those that died (at 0 on the y axis), with their antibody level on the x axis. The blue dashed line is the estimated logistic model relating antibody level to the probability of survival based on the data set. 

Subsequent studies should be designed with the aim of improving the precision of the fitted model. In the example shown, vaccine doses should be chosen such that resulting antibody levels include intermediate survival probabilities. In this example, the target would be levels between approximately 300 and 5000 antibody arbitrary units. Simulation techniques using pilot data can be used to guide the choice of vaccine doses in the study design that will use minimal numbers of NHP and still generate the optimal data set for robust logistic regression. When preliminary human data are available, these can be incorporated with a view to optimizing the estimation of vaccine efficacy in humans. When there are several candidate correlates of protection, receiver operating characteristic (ROC) curves can be created for each one to determine which one has the greatest predictive value. These provide an alternative to the logistic regression curve for visual and quantitative assessment of the strength of the relationship between the correlate and survival to support the choice of correlate.

In parallel with the NHP challenge studies, a clinical study is required to characterize the distribution of antibody levels achieved by the planned vaccine regimen in human subjects. The efficacy of the vaccine in humans is estimated by calculating the probability of survival for each human subject in the clinical study from their measured antibody level via the relationship estimated from the NHP challenge studies. These probabilities are averaged to give the vaccine efficacy estimate and a bootstrap confidence interval for the efficacy estimate [85,86,87].

The immunobridging approach outlined above is exemplified using the case studies below. Each case study differs somewhat in the animal model(s) and immune correlates selected for immunobridging. Just as there are unique aspects to each of these studies, so too will there be unique vaccine-specific challenges for every candidate. A solid foundation and precedent(s) will assist in surmounting such challenges. 

## 4. Immunobridging Case Study: BioThrax

The AVA anthrax vaccine was originally licensed in 1970 for pre-exposure prophylaxis (PrEP) against anthrax disease caused by the bacterium *Bacillus anthracis* [88]. In 2015, the FDA approved AVA for a second indication, post-exposure prophylaxis (PEP) of disease following *B. anthracis* exposure, when administered in conjunction with recommended antibacterial drugs. While the PrEP licensure was achieved using clinical trial efficacy data obtained at a time when there was still a significant number of anthrax cases in the U.S., the PEP indication was obtained under the FDA Animal Rule. This was the first vaccine to be approved for an indication under this regulatory pathway [28,89]. To date, AVA remains the only vaccine approved under the Animal Rule [90]. We have previously reviewed the FDA Animal Rule approval of AVA from a regulatory/procedural perspective [29]. Here, we review the establishment of the correlate of protection and the immunobridging analysis for AVA associated with its approval. Despite the large body of data that had been gathered in humans and animals prior to pursuing PEP licensure of AVA, additional studies were required to generate data to support approval for the new indication. In addition, a strategy to bridge the animal immunogenicity and survival data to the human immunogenicity data needed to be developed [3,89].

Two well-characterized animal models were used to obtain the data required to support PEP licensure of AVA. The rabbit (New Zealand white rabbits, *Oryctolagus cuniculus*) and NHP (cynomolgus macaques, *Macaca fascicularis*) models of inhalational anthrax were well characterized [91,92,93]. In 2002 and 2007, the FDA, the U.S. National Institutes of Health and the U.S. Department of Defense sponsored workshops to discuss how the Animal Rule would be used for anthrax vaccines [94,95]. Among other discussions, a consensus was reached that rabbits and NHPs are appropriate animal models to generate pivotal animal data for anthrax countermeasures, and that neutralizing antibody levels to the *B. anthracis* lethal toxin [64] generated by vaccination could serve as the correlate of protection [3,89,96]. Species-independent toxin-neutralizing antibody assays were available and validated, allowing for the direct comparison of the TNA data generated in rabbits and NHPs with that in humans, which was critical for immunobridging [3,91,92]. 

Numerous PEP, PrEP and passive transfer studies in NHPs and rabbits were conducted to support licensure. PrEP and PEP study data from both the rabbit and NHP models were evaluated to determine the relationship between vaccine-induced TNA titers and survival [3]. The data obtained from the various animal studies were used to support the premise that neutralizing antibodies to the *B. anthracis* lethal toxin correlate with protection, and that the level of neutralizing antibodies generated by immunization and measured immediately before lethal *B. anthracis* challenge is predictive of the probability of survival. Passive antibody transfer studies in rabbits and NHPs demonstrated this to be a mechanistic correlate of protection, since neutralizing antibodies alone were sufficient to confer protection against lethal *B. anthracis* challenge [97].

PEP studies were conducted with concomitant antibiotic treatment and immunization immediately following lethal *B. anthracis* challenge to demonstrate that AVA provided benefit over antibiotic treatment alone. At the time, the only approved treatment for *B. anthracis* exposure was 60 days of antimicrobial treatment [3]. However, the PEP challenge model could not be used to bridge to human immunogenicity data because the antibodies induced by *B. anthracis* challenge confounded the ability to measure similar antibodies generated at the same time by immunization with AVA. The FDA agreed with the AVA developer that the PrEP rabbit and NHP models of inhalational anthrax were appropriate for determination of the threshold of protection, as the immune responses can be assessed in relation to immunization alone [3]. 

Immunogenicity data were used in a logistic regression analysis to estimate the probability of survival associated with various circulating TNA titer levels immediately before challenge. A TNA value on day 69 (rabbits) or 70 (NHPs) immediately before *B. anthracis* aerosol challenge, which was associated with a high probability of survival, was identified [3]. The TNA threshold was bridged to human post-vaccination immune levels on day 63 after the first immunization. This time point corresponds to 3 days after a 60-day antimicrobial treatment would have ended [3,89]. Using this methodology, an estimated 82% of the human subjects had a pre-challenge TNA value at or above that correlating with 70% survival in rabbits and 88% survival in NHPs [3]. 

Licensure of AVA for PEP illustrates the complexities of the Animal Rule licensure pathway. For PEP licensure, significant time was devoted to exploring the utility of each of the different animal models and then determining the most appropriate methodology for bridging and estimating efficacy in humans. Although animal models and immunoassays had already been developed and approved for use by the regulatory agency, completing all activities required to support PEP licensure took approximately 10 years. AVA put into practice the Animal Rule regulations for the first time, establishing a scientific framework for approaching licensure via this pathway, revealing how critical communication with the FDA is when building an Animal Rule licensure plan. As the regulator, FDA concurrence was critical for selection of the animal models, study design and selection of the immune correlate of protection. 

## 5. Immunobridging Case Study: Zabdeno, Mvabea

Janssen Vaccines and Prevention B.V. developed a two-dose vaccine regimen (Zabdeno, Mvabea) for prevention of Ebola virus disease which was approved by the European Medicines Agency without human efficacy data [5]. Here, we review published data described in Roozendaal et al. that laid the foundation for licensure [2]. The work offers a filovirus-specific case study in identification of an immune correlate of protection as well as immunobridging. While similarities between this case study and that of AVA can be drawn, distinctions can also be made, emphasizing the importance of vaccine-specific strategies to obtain licensure in the absence of human efficacy data. 

Roozendaal et al. examined several potential immune correlates of protection, their relationships to survival and their ability to discriminate between survivors and nonsurvivors [2]. All studies used cynomolgus macaques challenged with a target dose of 100 plaque forming units (PFU) of EBOV Kikwit, equivalent to at least 200 times the lethal dose as shown by the authors in the context of their work [2]. The challenge was 100% lethal in all control animals. Blood was collected prior to virus challenge to examine pre-challenge immune levels with three bioassay methods, including ELISA to measure anti-EBOV GP serum antibody binding concentrations, pseudovirus neutralization assay (psVNA) to measure neutralizing antibody titers and interferon gamma ELISpot on peripheral blood mononuclear cells (PBMCs) to indirectly measure GP-reactive T cells. In regimen finding experiments, prime (Zabdeno)-boost (Mvabea) intervals were evaluated with a limited variation in vaccine dose. A dose interval of 8 weeks resulted in the highest immunogenicity and 100% protection. Regimens with shorter dose intervals resulted in less than 100% survival [2]. 

Roozendaal et al. used data from all regimens, including those with dose 1 and dose 2 separated by less than 8 weeks, to develop the logistic regression model by correlating each immunological parameter with survival. Anti-GP binding antibody concentrations, neutralizing antibody titers and GP-reactive T-cell spot counts were graphed separately against survivors and nonsurvivors [2]. All immune parameters showed significant and positive correlations with survival [2]. ROC analysis revealed that GP-reactive T cells showed the least significant correlation with survival among the three parameters with the lowest area under the curve (AUC, 0.710), while the highest AUC (0.880) was neutralizing antibody titers, although AUCs for neutralizing antibody titers and binding titers were similar [2]. Based on these findings, the authors elected to use anti-GP antibody binding concentrations as the correlate of protection for immunobridging analysis, because the ELISA is a more robust and reliable assay than the psVNA [2]. 

To refine the logistic regression model with anti-GP binding antibodies, dose reduction studies were performed. The additional studies resulted in a similar logistic regression model; however, the AUC of the ROC curve further improved. In other words, the ability to discriminate between antibody concentrations of survivors and nonsurvivors improved. For this vaccine, anti-GP binding antibodies were determined to be the primary factor predicting the outcome of the vaccine regimen because additional parameters such as dose level did not have an additive effect on the logistic model. Through immunobridging analyses of Phase 1 human immunogenicity data and the logistic model (representative of the relationship between anti-GP binding antibody concentrations and survival in NHPs), it was determined that the levels of vaccine induced anti-GP antibodies in human subjects predicted about 48% survival based on the NHP immune correlate of protection. Immunobridging was facilitated by having the same validated ELISA for humans and NHPs to determine anti-GP binding antibody concentrations in both species. Indeed, parallel NHP and human serum dilution curves in this assay enabled a direct comparison of ELISA results. Although direct comparison was possible, Roozendaal et al. reasonably concluded that the roughly 48% predicted survival probability in humans likely represents a conservative estimate of the protective effect in humans due to the stringency of the NHP challenge model [2]. 

This example demonstrated successful use of a single well-characterized NHP model for vaccine regulatory approval based on animal survival data and immunobridging. Irrespective of whether binding antibodies are mechanistically involved in protection, it was shown that anti-GP binding antibodies positively correlated with survival and could be successfully used to discriminate between survivors and nonsurvivors in the context of this vaccine. Finally, these studies are a reminder that, while mechanism is important, a clear mechanism-linked immune parameter is not required for the correlate of protection.

## 6. Conclusions 

We have herein covered some of the key considerations important to developing an immunobridging strategy for vaccine licensure via the Animal Rule. Appropriate selection of an animal model(s) that reflects human disease for the pathogen of interest and establishment of an animal immune correlate of protection in the selected model that is relevant and assayable in humans are essential to ensuring the feasibility of immunobridging. Once the animal model(s) and the immune correlate of protection have been established and the effective vaccine dose and regimen have been determined, dose reduction studies are performed to build a logistic model to define the relationship between the value/titer of the immune correlate of protection and survival at each dose. Creative strategies may be required to populate the logistic model effectively across the range of protection such as the reduction of time between prime and boost for a two-dose vaccine regimen. Onset and duration of protection studies may be done to inform potential real-world use of the vaccine. For instance, durability of protection studies may assist in inferring human vaccine protection months post-vaccination (in what may be a more realistic exposure scenario than the 4-week post-vaccination challenge studies often done in NHPs) and may inform regarding how the amnestic response may contribute to protection. 

In addition to reviewing the components required to build an immunobridging strategy during the KOL meeting, there were important filovirus-specific takeaways. Meeting participants generally agreed that NHPs display key aspects of human filoviral disease pathophysiology and, therefore, represent a sufficiently well-characterized model for filovirus countermeasure and therapeutic development, a critical criterion to satisfy the Animal Rule. Agreement on this topic with regulators could reduce the need for preliminary work on the selection of a filoviral animal model(s). 

Some topics herein presented will benefit from future discussion. For instance, we have not focused on clinical trial design, where parameters such as dose selection, clinical endpoint definitions, statistical analysis measures and tools are important topics of discussion since trial design is critical to the success of an immunobridging strategy. Finally, consideration should be given to mechanisms to license vaccines based on animal efficacy data in countries where the pathogen is circulating, such as those on the African continent (in the case of filovirus vaccines), where there is greatest need. Regulators are engaged in these discussions and are helping to establish solutions that will be acceptable for a licensure package. These discussions can and should be part of larger conversations to promote the adoption of licensure pathways that permit the inclusion of animal survival data in the absence of human efficacy data when human efficacy data is unattainable. Such pathways would be a boon to vaccine licensure and, ultimately, public health.

## Data Availability

Not applicable.
